# Environmental and Economic Analysis of FDM, SLS and MJF Additive Manufacturing Technologies

**DOI:** 10.3390/ma12244161

**Published:** 2019-12-11

**Authors:** Vincenzo Tagliaferri, Federica Trovalusci, Stefano Guarino, Simone Venettacci

**Affiliations:** 1 Department of Enterprise Engineering, University of Rome “Tor Vergata”, Via del Politecnico, 1-00133 Rome, Italy; federica.trovalusci@uniroma2.it; 2 Engineering Department, University “Niccolò Cusano”, Via Don Carlo Gnocchi, 3-00166 Rome, Italy; stefano.guarino@unicusano.it (S.G.); simone.venettacci@unicusano.it (S.V.)

**Keywords:** additive manufacturing, fused deposition modeling, selective laser sintering, multi-jet fusion, life cycle assessment

## Abstract

In this study, the authors present a comparative analysis of different additive manufacturing (AM) technologies for high-performance components. Four 3D printers, currently available on the Italian national manufacturing market and belonging to three different AM technologies, were considered. The analysis focused on technical aspects to highlight the characteristics and performance limits of each technology, economic aspects to allow for an assessment of the costs associated with the different processes, and environmental aspects to focus on the impact of the production cycles associated with these technologies on the ecosystem, resources and human health. This study highlighted the current limits of additive manufacturing technologies in terms of production capacity in the case of large-scale production of plastic components, especially large ones. At the same time, this study highlights how the geometry of the object to be developed greatly influences the optimal choice between the various AM technologies, in both technological and economic terms. Fused deposition modeling (FDM) is the technology that exhibits the greatest limitations hindering mass production due to production times and costs, but also due to the associated environmental impact.

## 1. Introduction

Interest in additive manufacturing (AM) is increasing rapidly in many technology domains (Bandyopadhyay and Heer 2018 [1]). Currently, AM processes allow for the production of customizable three-dimensional (3D) structures with different accuracy levels depending on the selected technology (FDM, SLS, MJF, SLA, etc.), in an entirely automated manner, producing low waste and avoiding the need for supply chain integration (Eyers and Potter 2017 [2]; Kruth, Leu, and Nakagawa 1998 [3]). These processes utilize computer aided design (CAD) information to produce 3D objects through successive superimposed layers of material. The materials used for AM vary widely, from polymers to ceramics, and from composites to metals (Bourell et al. 2017 [4]). They can be powder-based, in the form of liquid resin, or solid filaments. The choice depends on the individual technique and the application of the end product. The production of 3D structures with complex geometries by AM has recently been applied in different fields, including biotechnology (Bose et al. 2018 [5]) and medicine, e.g., to model anatomical structures in neurosurgery (Randazzo et al. 2016 [6]; Scerrati et al. 2019 [7]), to model complex molecules and protein interactions (Włodarczyk-Biegun and del Campo 2017 [8]; An et al. 2015 [9]), and to fashion customized laboratory tools (Murphy and Atala 2014 [10]; Berman 2012 [11]; Ju et al. 2014 [12]); racing (Nickels 2014 [13]; Harrys and Halliday 2013 [14]), jewelry (Fabian 2016 [15]), and electrical components (Kwok et al. 2017 [16]; Flowers et al. 2017 [17]; Mu et al. 2017 [18]).

However, the choice of materials can be limited in terms of durability/strength of resulting products (Guo and Leu 2013 [19]). The sizes of the objects produced are also limited by the size of the 3D printer currently available on the market. Other disadvantages are related to surface finishing, which can be compromised by the layering process. Many studies are aimed at developing finishing processes suitable for complex geometries, such as abrasive fluidized beds (Atzeni et al. 2016 [20]; Barletta and Guarino 2010 [21]). Moreover, 3D printer production volumes are very limited compared to mass production processes. In addition, the processes of powder fusion have much higher energy consumption (for a raw material mass unit) than traditional processes (Wong and Hernandez 2012 [22]; Yoon et al. 2014 [23]).

The AM technologies that were analyzed in this work are selective laser sintering (SLS), fused deposition modeling (FDM), and multi-jet fusion (MJF). FDM is a common extrusion process in which the material passes through a nozzle, where it is heated, melted, and deposited layer by layer. Commonly, the nozzle can move horizontally, and the platform moves vertically after the deposition of each new layer, in some FDM technologies, the nozzle can move both horizontally and vertically. FDM is a commonly used technique for many low-cost 3D printers, even for home use. This technology allows for working with many other plastic materials in addition to nylon and PC, making it easily accessible and able to produce objects with good structural properties that are very close to the final product. Its accuracy and speed are low compared to other processes, and the accuracy of the final object is limited by the thickness of the nozzles.

Selective laser sintering (SLS) belongs to the class of powder bed fusion (PBF) processes, techniques that use a laser or an electron beam to melt and bond powdered material. Laser sintering machines consist of three components: a heat source to melt the material, a method to control the heat source and a mechanism to add new layers of material to the previous ones. The SLS process has the great advantage of not requiring any additional support structure, as the powdered material provides adequate support to the object throughout its construction process. Different materials commonly used for SLS processes include plastic, metal, glass, ceramics, and composites (Kruth et al. 2003 [24]; Parandoush and Lin 2017 [25]).

Multi-jet fusion (MJF) is a very innovative method developed by Hewlett-Packard (HP). It is based on the use of numerous material jet nozzles that stratify various liquid agents on the printing plane.

The different AM technologies mentioned above were compared, and a life cycle assessment (LCA) was developed to verify that these technologies are advantageous in terms of every aspect of environmental impact. The LCA methodology is based on the evaluation of each phase of the life cycle of a product to allow for the assessment of the potential impact on human health, on the quality of the ecosystem and on the exhaustion of the planet’s resources. In this study, the analysis was conducted by means of a computerized tool, SimaPro (PRé Sustainability, Stationsplein 121, 3818 LE Amersfoort, The Netherlands), which allows for the implementation of an LCA model, from which it is possible to extrapolate environmental effects (Guarino et al. 2017 [26]).

The results demonstrate that there are significant differences between AM technologies, not only in relation to production costs but also in terms of the eco-compatibility of each production technology.

## 2. Materials and Methods

### 2.1. Experimental

There are a wide range of AM technologies for constructing an object layer-by-layer. In particular, in this work, selective laser sintering (SLS), fused deposition modeling (FDM) and multi-jet fusion (MJF) were analyzed and compared. The printers reported in Table 1, which are already on the market, corresponding to the technologies in question, were chosen. All the technologies were studied, and for one typology, two machines were considered.

These printers were selected to develop different types of components. Naturally, the first fundamental phase consisted of the selection of components with dimensions compatible with the various working chambers of the printers. Approximately, each of the four 3D printers has a working chamber of 0.027 m^3^ (300 × 300 × 300 mm); thus, the objects to be made must necessarily fit within those dimensions.

**Figure 1:**
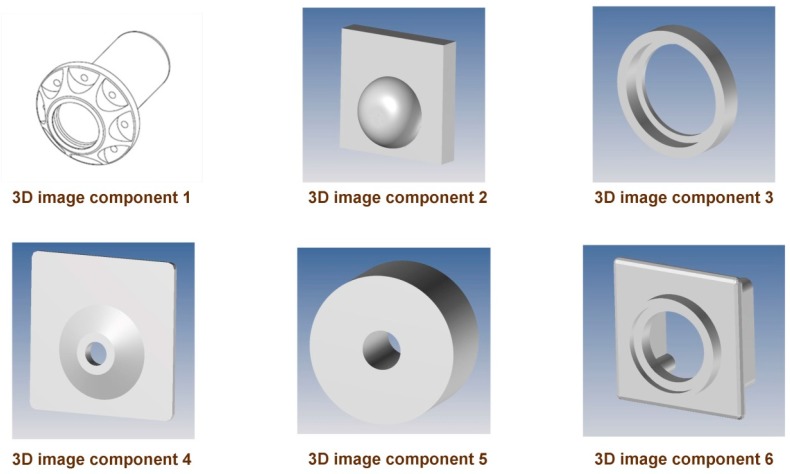
Geometries of the six components analyzed.

The authors decided to select six different objects with different geometries and with a volume ranging between 350 mm^3^ (component 4) and 100,700 mm^3^ (component 1). Figure 1 shows images of the six components.

Naturally, every printer must use its own materials, with technical characteristics that can vary considerably. To ensure that the results of the analysis of the various machines and technologies would be as comparable as possible, it is necessary that the materials chosen to realize the single component with the various machines are as similar as possible: for this reason, Polyamide 12 (or, more commonly, Nylon 12) was chosen for all printers, in the form of powder for SLS and MJF and filament for FDM.

The Fortus 450mc printer builds the pieces by overlaying layers from bottom to top by heating and extruding the thermoplastic filament. The process is simple and involves a succession of predefined phases (Bikas et al. 2016 [27]):

- Preliminary operations: the 3D CAD file is decomposed into layers and positioned to calculate the filament extrusion path, in addition to any supporting materials needed;
- Production: the 3D printer heats the thermoplastic to obtain a semi-liquid state and deposits it in tiny drops along the extrusion path. If necessary, the 3D printer deposits a removable material that serves as a support structure;
- Post-processing: the user removes the support material or dissolves it in water and detergent, such that the piece is ready for use.

The volume of the printer’s construction chamber is about 0.0585 m^3^ (406 × 355 × 406 mm or 16 × 14 × 16 inches). It is equipped with two individual housings for the stratification material and for the support. The latter is soluble for most materials, removable for PC-ISO and ULTEM, and soluble or removable for PC (Bikas et al. 2019 [28]).

The weak point of this printer, and more generally of all FDM technology, is the accuracy that can be obtained: the thickness achievable ranges from a minimum of 0.127 mm (for simpler materials) to a maximum of 0.33 mm (for materials with more advanced features).

The sPro 60 printer is an SLS series machine built by 3D Systems. It is a printer that uses selective laser sintering technology, namely, laser sintering of a powder bed (powder bed fusion). The printer uses a carbon dioxide (CO_2_) laser to melt small particles of plastic powder into a desired three-dimensional shape. After scanning each cross section, the powder bed is lowered by a height equal to the thickness of a layer.

This 3D printer produces functional thermoplastics with high-quality resolutions and surface finishes, whose fields of potential application are many, ranging from industrial design to medical devices to components for pipelines to custom devices for patients. However, the sPro 60 printer does not allow for creating an empty, completely closed object because the non-sintered powder inside the object cannot be discharged. Moreover, this printer is compatible only with the material supplied by 3D Systems, which is subject to a registered trademark.

The EOS P396 printer also belongs to the selective laser sintering technology category, so the operating principle is the same as that of sPro. The most common material used in all SLS machines is polyamide (PA), typically known in its synthetic form, nylon.

The final printer considered is the Jet Fusion 3D Printer produced by HP. This printer has revolutionized the 3D printing market, introducing a new construction mode, multi-jet fusion. The Jet Fusion printer uses an array of 30 jet nozzles (which can release up to 350 million drops per second at the maximum print resolution) that stratify various liquid agents on the printing bed. A major weakness, however, is the scarcity of usable materials: at the moment, this printer uses only two types of material with a fine-grained polyamide base.

### 2.2. Economic Model

Among the different materials that can be processed by each machine, it was decided to select polyamide 12 (nylon 12), as this type of material can be used with all of the selected printing alternatives. This thermoplastic material was particularly suitable for the production of functional prototypes, production equipment and finished parts. The amount of reusable material was approximately the 80% of the total that was not used in the construction of the piece, whereas the remaining 20% was discarded because it was compromised by the production process. Only in the FDM technology printer, at the end of the process, was there no excess material to be recovered. There was also an average volumetric shrinkage of approximately 2% for all the evaluated printers; this value was therefore used for calculating the appropriate powder bed level.

To create an effective economic model, the authors decided to set as targets the determination of both the maximum number of pieces that can be produced in one year (that is, the annual production capacity) and the cost incurred for each single component produced.

First, it was necessary to determine the time the laser (or the lamp in the case of the HP-MJF printer) would need to sinter the powder to construct the object. Therefore, starting from the quantity (in volume) of the necessary powder and the nominal speed, communicated by the constructor, the effective processing time and the annual production capacity were obtained. For its determination, assumptions about the working conditions of the printers were made. Given these assumptions, the various costs (the cost of raw materials, labor costs, energy costs, and extra costs related to the specific printer) were calculated; added together, these yielded the cost incurred to produce a single component.

To better delineate the procedure that was followed in the development of the economic model, when comparing the various technologies, it was decided to describe a single case study, that of component 1 for the EOS P396 printer. The internal volume of the printer chamber was about 0.0694 m^3^ (340 × 340 × 600 mm), while the component 1 had an overall length of 130.5 mm and a maximum external diameter of 97 mm.

**Figure 2:**
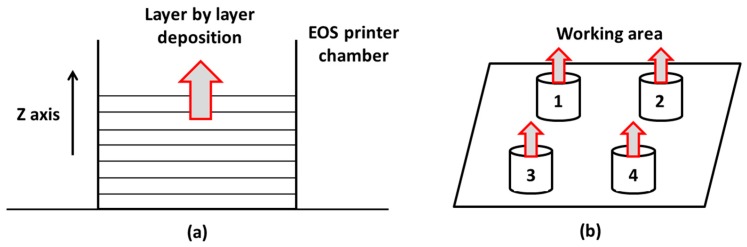
Layer by layer deposition technique (**a**); qualitative arrangement of component 1 in the EOS printer chamber (**b**).

The SLS (selective laser sintering) printer involves the deposition of a powder bed in a construction chamber, filled with inert gas (nitrogen), and layer-by-layer sintering of the powder using a CO_2_ laser. The component n.1 had a shape similar to that of a screw. The process is calibrated on the largest possible batch to be built, consisting of 4 units, according to Figure 2, that shows the layer by layer deposition technique (a) and an overview drawing of the arrangement of component 1 inside the chamber (b). 

The starting point is the calculation of the height that the dust (deposited each time after the laser beam scans) has to reach.

Each piece is built in a vertical position, with the base of the cylindrical part resting on the construction plane and the part with the shape of a truncated cone at the top, to obtain a simpler and more precise laser processing and to realize in a short time even more components at the same time. The Fortus 450mc printer (and, in general, every FDM technology 3D machine), on the other hand, can work with only one component at the same time, resulting in a significant increase in processing time if more than one object is to be made in the same production process (as in a batch production process).

According to the EOS statement, the maximum construction speed of the P396 is 48 mm/h, expressing in millimeters the height of the powder bed necessary to realize a single component. Knowing both the maximum construction speed and the height reached by the powder bed, depending on the size of the 3D object to be created, it is possible to determine the minimum time needed by the EOS P396 printer to build the component, which is approximately 167 min. Therefore, to produce a lot of four units, it will take approximately 668 min.

For the calculation of the total time of a processing cycle, additional time for further activities, necessary for the production, has to be considered in addition to the lot effective processing time already calculated. These are preliminary activities for preparing the printer (45 min, also including the time necessary to fill the working chamber with nitrogen) and activities subsequent to printing, i.e., for recovery of dust in the working chamber and cleaning the finished pieces (45 min) and for printer cooling at the end of process (60 min). Thus, a total time of 150 min is obtained for related machining activities. By considering this additional time, the cycle time for producing a batch of component 1 becomes 818 min, or 13.63 h.

In the context of a cost assessment, it is also considered that the 3D printer, inserted into a production process, works 200 days per year in two 8-h shifts. The printer needs a very small monthly maintenance intervention, estimated (conservatively) to be approximately 8 h every month. Thus, the hours in which the 3D machine is used for processing is given by the opening hours of the plant (3200 h/year) minus the hours dedicated to maintenance (96 h/year), or 3104 h/year. Starting from the operating time, an annual production capacity of 892 components per year has been calculated for the EOS P396, assuming four components in the production chamber and a defect rate equal to 2%. Moreover, it has been considered that all defective parts are discarded.

Table 2 reports the quantities of powder that must be inserted inside the chamber for the production of a single component or a lot.

On the basis of the values reported in Table 2, it is evident that it is definitely more convenient to make the EOS P396 printer work on several objects at the same time in the same working cycle rather than on a single object.

The following cost categories will be considered in the economic analysis:

- Raw materials, which are necessary both for the finished product and for the support;
- Labor, which is necessary for the correct operation of the printer;
- Energy, which is necessary to power the printer for all its functions.

Considering the raw material, PA2200 has a cost of approximately 77 €/kg (if sold in 20-kg lots). Based on the amount of material necessary for the production of a single component, reported in Table 2 (44 g) and considering the possibility of re-using the powder not compromised by the process, it is possible to determine the expense to be incurred for the purchase of the raw material. On average, the percentage of dust that remains in the working chamber and can be used again for other processes is approximately 80%. On the basis of these assumptions, a value of 4.24 €/piece was obtained for the cost of raw materials.

This printer also requires a manpower contribution. Thus, costs incurred for manpower to use the EOS P396 printer must be considered in the calculation of the production cost of a single component.

For the correct operation of the printer, only one operator is required for every shift; thus, every day, two operators were present. Their annual salary was assumed to be € 25.000. Considering 200 days/year, 8 h/day and 60 min/h, the product is 96.000 min/year of work for each operator at a pay rate of approximately 0.26 €/min.

Once they are started, the printers work automatically, so the operator’s contribution to the printer is limited to the preliminary setup and to the subsequent cleaning of the work chamber and post-processing operations on the components (the cooling process of the machine is automatic). All of these operations, as previously assumed, require a total duration of approximately 90 min for the production of a single lot.

The cost of labor is thus € 23.4 for these activities, or 5.85 €/piece, considering the allocation among all the components of the lot.

The last cost item to be considered is that related to energy consumption. The EOS P396 printer typically requires a nominal electrical power of 10 kW.

Assuming a constant electricity consumption equal to the nominal value and a cost of approximately 0.06 €/KWh, and taking into consideration only the time in which the printer actually works for the sintering of the powder material (equal to 668 min, or approximately 40.1 h), the energy cost is approximately 6.68 €/lot, or 1.67 €/piece. So the total production costs for a single component 1 built by EOS P396, assuming there are four components in the production chamber, are about 11.76 €.

The same analysis illustrated for component 1, built by the EOS P396 printer, was also repeated for the other three printers (six versions total, considering two versions analyzed for the HP Jet Fusion and three for sPro 60). For all selected printers, the maximum production batch that can be produced was four units of component 1.

For the HP and 3D Systems printers (namely, the sPro 60 family), the construction speeds considered in the analysis are the following: 2800 [cm^3^/h] for the HP Fusion 3D 3200, 4000 [cm^3^/h] for the Jet Fusion 3D 4200, 900 [cm^3^/h] for the sPro 60 SD, 1000 [cm^3^/h] for the sPro 60 Base HD, and 1800 [cm^3^/h] for the sPro 60 HD.

The construction speeds were very high for both versions of the HP printer but lower for all versions of the 3D Systems printer. After determining the volume occupied by a single component 1, it was easy to calculate the processing time. For the Fortus 450mc printer, the required processing time was provided directly by the machine once the component CAD file was inserted.

For the calculation of the annual production capacity, the same considerations made for the EOS P396 were made for all printers. The only exception is the fact that the other 3D machines do not work in an inert atmosphere: this leads to a shorter time (20 min for both the HP Jet Fusion 3D and the sPro 60) for the preparation of the printer before processing. For the Fortus 450mc, which does not work with dust and therefore does not require cleaning the work chamber after processing, the total additional time (including both preliminary and subsequent activities) is 100 min (60 of which are only for the machine cooling).

Then, the material cost items were calculated, as was performed for the EOS P396, starting from the price for one kilo of the powder (for powder bed fusion machines) and the weight of the powder itself. For the HP High Reusability PA 12 powder, an average price of 67.5 €/kg was considered, to which was added the cost of 4.7 €/piece related to the fusing agent and detailing agent, used by multi-jet fusion technology to create the material solid layer and to realize the details of the layer profile.

The price of Duraform PA powder (3D Systems material) is obviously higher, approximately 135 €/kg, mainly due to the superior mechanical properties of the material. On the other hand, no additional chemical agent is required for processing. Stratasys, instead, has stated that producing component 1 on the Fortus 450mc printer requires a cost of 18.97 €/piece and an additional cost of 7.53 €/piece for the support material (soluble and therefore easily removable at the end of the process).

The cost items for labor and energy must then be added to the total cost for the individual component. In particular, to calculate energy costs, the different energy consumptions of the printers must be considered: the nominal electric power is 10 kW for the HP Jet Fusion 3D 3200/4200, 7 kW for the Fortus 450mc and 12 kW for the three versions of the sPro 60. In all the cases, constant consumption of electricity equal to the nominal value during the processing time was assumed.

All the results obtained for component 1 were added to those obtained from the other case studies, obtained by combining each of the remaining 5 components for the selected printers. Aggregating these results, it was possible to compare various printing solutions, the results of which are reported in the appropriate section.

### 2.3. Life Cycle Analysis

In addition to setting up an economic model, an environmental impact assessment associated with the various AM technologies in question was carried out.

This environmental assessment was performed using the SimaPro v.7.1 computer software to implement an LCA (life cycle assessment) model. This method involves analyzing the entire life cycle of the developed product in detail, starting from the production cycle up to the disposal phase. Therefore, in this analysis, the authors considered not only the environmental effects of the production process but also the raw materials supplying phase, the use of the product and its final disposal.

The LCA method is regulated by the series ISO-14040 standards and provides for the definition of four main phases:

- Scope and objective definition;
- Inventory analysis;
- Impact estimate;
- Interpretation.

The first step is to define the component to be analyzed for the impact assessment. It was decided to analyze a 4-unit batch of component 1, evaluating both performances for each machine, and then the input and output flows connected to the realization of the lot in question.

In particular, in defining the different LCA models associated with each of the various technologies analyzed, it was decided to consider the following flows, associated with a single-lot production process:

- Material: raw material, support material, chemical agents, etc.;
- Energy: compressed air, electricity;
- Disposal: excess powder, finished product;
- Recovery and rework of the excess raw material.

In the definition of the LCA models, some simplifying hypotheses were made. The PA 6 was assumed to be raw material for all the processes instead of PA 12 (not present in the SimaPro database); since the properties of two materials are very similar, this hypothesis should not strongly alter the results and considerations of the LCA.

A second assumption about the electricity consumption of AM machines was made: having no concrete results regarding the real consumption of printers for the realization of a four-unit batch of component 1, built using the PA 6 material, constant electricity consumption equal to the nominal value reported in the technical sheet of each printer was assumed.

In the context of each of the components under examination, not knowing their future use and their useful life, the phase relative to the useful life of the component was neglected, focusing instead on the production and disposal (or recycling) phases of the object at the end of its life. 

**Figure 3:**
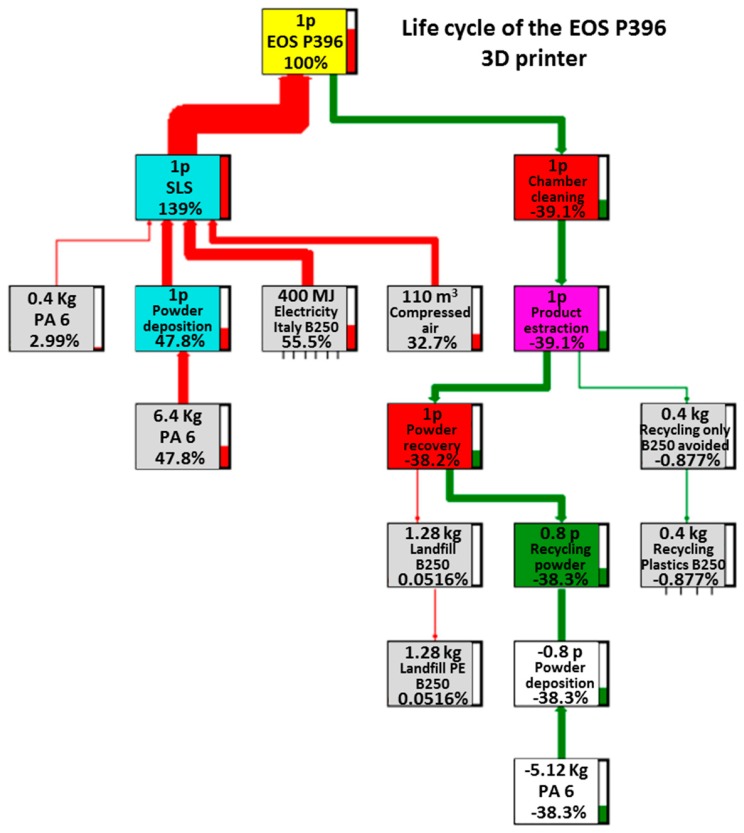
Life cycle of the EOS P396 3D printer.

**Figure 4:**
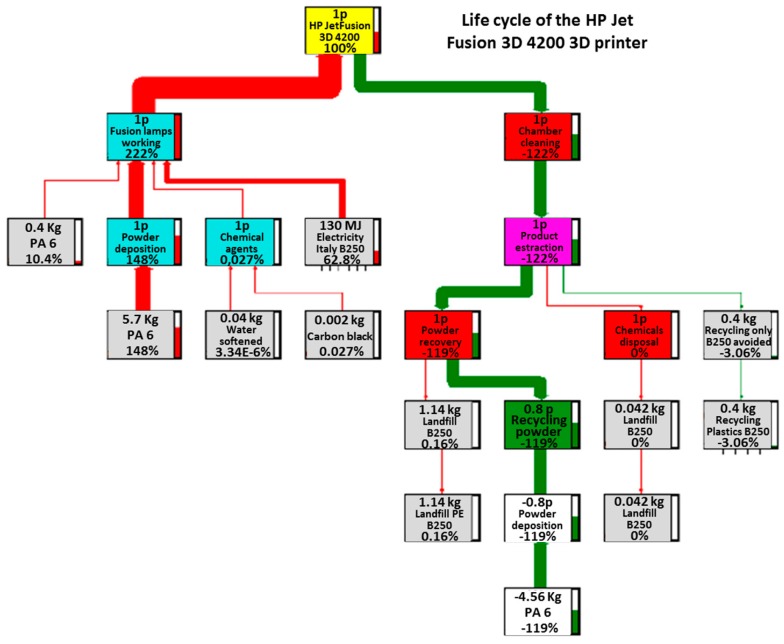
Life cycle of the HP Jet Fusion 3D 4200 3D printer.

**Figure 5:**
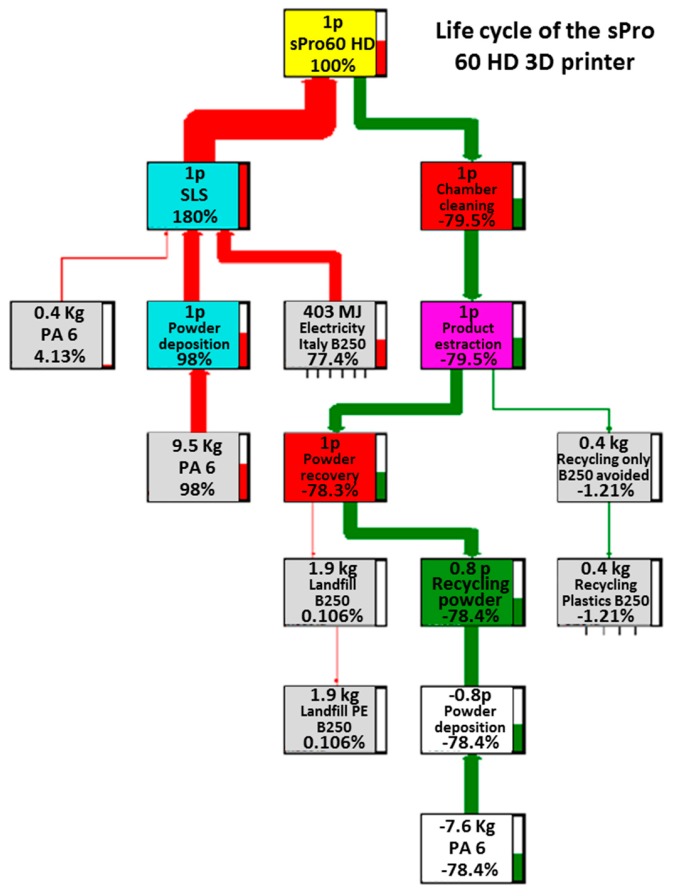
Life cycle of the sPro 60 HD 3D printer.

**Figure 6:**
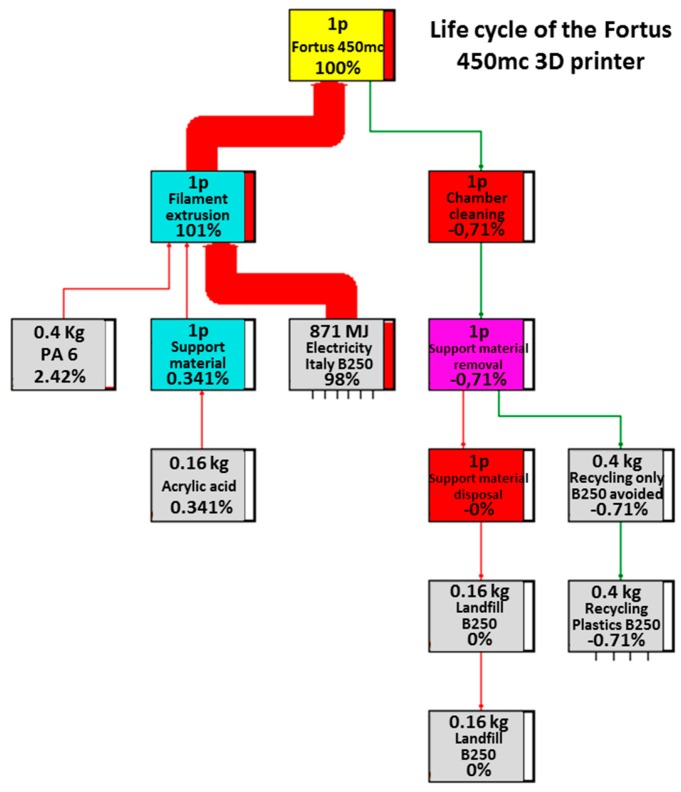
Life cycle of the Fortus 450mc 3D printer.

The four main trees related to the processes under examination are reported in Figure 3, Figure 4, Figure 5 and Figure 6. These figures report numerically all the input and output flows, associated to the different AM technologies analyzed, numbers which the SW needs to calculate the environmental impacts related to the different impact categories (better described in Section 3.2). Note how, for each of the processes in question, the percentage impacts related to the different input and output streams have been calculated, according to Eco-Indicator 99 method, developed by Pré Consultans BV, Netherland (Goedkoop et al. 2013 [29]). This method involves converting the inventory data into contributions related to the impact categories, multiplying them by the related characterization factors. These values, to be able to be compared with each other, need to be normalized first (i.e., made homogeneous, because of the different measurement units for the categories) and then weighted by a weight factor (associated with the calculation method) and finally added to obtain a single impact indicator associated with the product (called “cumulative impact” or also “single score”).

It is possible to notice that in the case of FDM technology, the lone substantial flow was that associated with the electric current consumption. In fact, the contributions of both the basic and the supporting material were significantly reduced.

In contrast, for SLS technology machines, the contributions associated with energy flows and raw material consumption seemed to be comparable with each other. In particular, for the sPro 60, the greatest contribution in the final assembly is that due to the support material (98%), which also exceeds that of the electric current (77.4%). However, there is an important difference between them: part of the support powder is recovered in the disposal phase, thus generating a recovery of 78% of the total contribution and greatly reducing the impact of the support material (to approximately 20%), something that does not happen with the electricity consumption, which will continue to account for almost all of the remaining 80%.

However, a similar situation occurs for the EOS P396, in which there is also an important contribution associated with the use of compressed air, which reduces the impact of electricity consumption to just over 50%, with the remaining part (approximately 10%) associated with the impact of the raw material consumption, both for basic and support material, after its recycling.

Finally, for the HP Jet Fusion, it is possible to notice much lower energy consumption is than for other additive manufacturing machines; however, this consumption accounts for over 60% of the total overall impact, thus indicating a possible lower impact potential of this technology on the environment compared to all of the others studied. The remaining fraction of the total impact is always due to the raw material consumption, both as a support and as a basic material for the realization of the finished object; this share is always considerably reduced due to the recycling of 80% of the total powder needed for the process.

## 3. Results and Discussion

### 3.1. Economic Analysis

The first comparison that can be made concerns the maximum number of pieces that can be produced within one year. To calculate this value, it is necessary to consider that the same hypotheses (about plant opening days, daily working shifts, and faults) are valid for all printers to obtain the most comparable results possible.

On the other hand, regarding the lot dimension to be examined for the individual components, it was considered necessary to construct the largest possible batch, while always respecting the dimensional constraints of the working chamber, to reduce the cycle time as much as possible and consequently increase the number of workable pieces. For component 1, as previously noted, the lot was the same for all types of printers, but it is not the same for all components. For example, for component 4, in addition to component 5, the considered lot consists of six units for the HP Jet Fusion 3D 3200 and 4200 printers, nine units for the EOS P396 and sPro 60 printers (SD, Base HD, and HD-HS), and 12 units for the Fortus 450mc.

**Figure 7:**
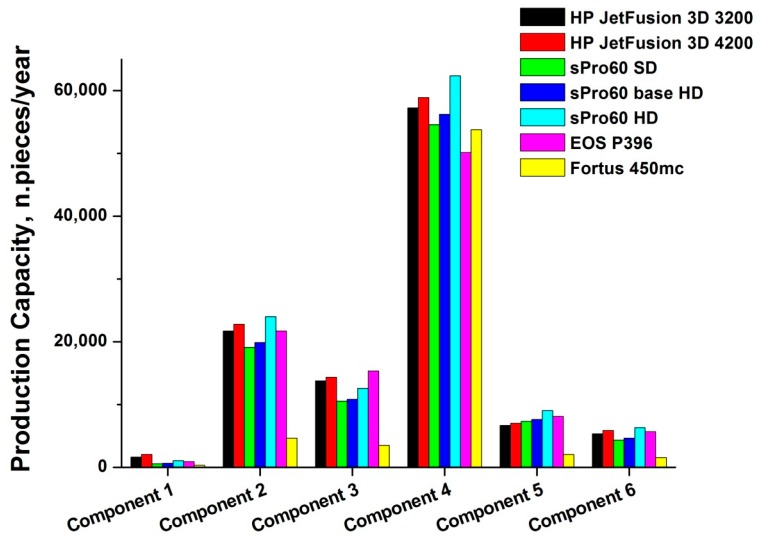
Annual production capacity of the various 3D printers for every component examined.

The production capacity values obtained for all types of printers studied and the various components analyzed are plotted in Figure 7. The maximum production capacity is always reached for the production of component 4 (more than 62.000 units produced by the sPro 60 HD printer), whereas the lowest values are always found for the production of component 1, which exceeds 2.000 units per year only for the HP Jet Fusion 3D 4200 printer.

Figure 7 clearly shows that the Fortus 450mc printer is always characterized by lower production capacity, with the exception of component 4, for which the results for all 3D machines are similar. This result basically indicates how the Fortus 450mc is in fact limited by poor performance in terms of the number of parts that can be built, compared to the other printers. Considering also that the working chamber of the Fortus 450mc is the largest among those analyzed (having a volume of about 0.0585 m^3^), this result shows even more clearly how the Fortus 450mc is the least suitable solution for production, due to its inability to build multiple objects at the same time. The Fortus 450mc, similar to other FDM technology printers, in fact has only one extruder nozzle, which takes care of melting the filament of raw material and depositing the drops of molten material on the construction platform.

It is also interesting to evaluate the cost of materials for each of the cases analyzed. The overall results are presented in Table 3 for each of the analyzed case studies.

Based on the unit costs and required quantities, the highest purchase cost of materials is required by the Fortus 450mc printer, for each built component, whereas the printer that requires the least cost is the EOS P396. This result is strongly affected by the cost of the raw material, which is significantly higher for the FDM printer compared to both the SLS and MJF machines, in addition to the different production capacity among the 3D machines under examination, depending on the size of the chamber, which entails a different batch size (Gibson, Rosen, and Stucker 2010 [30]; Costabile et al. 2017 [31]).

**Figure 8:**
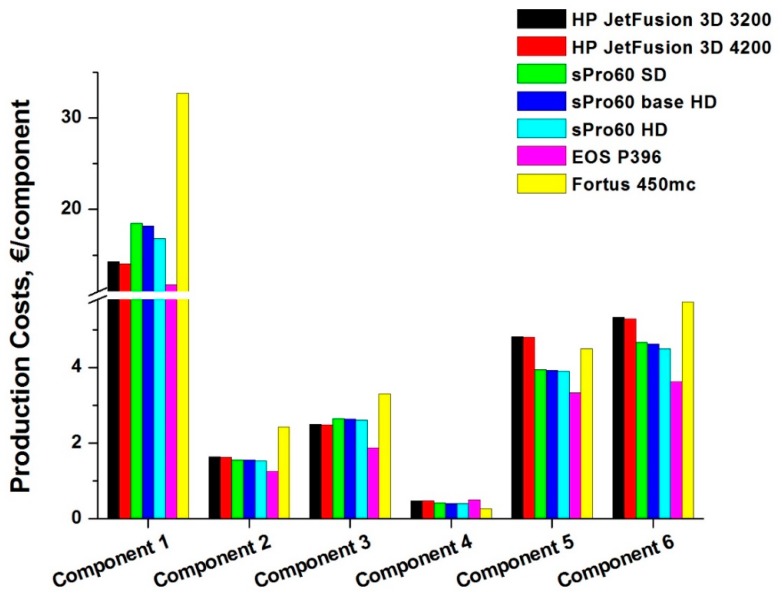
Total production costs for each component/printer case study.

Figure 8, instead, shows the total costs incurred for the production of a single component belonging to a production batch. Analyzing the total costs for each component, it is possible to observe clearly how the EOS P396 printer is the one characterized by the lowest costs, for each of the components under examination, except for case 4, where the Fortus 450mc has the lowest cost, whereas in all other cases analyzed, the FDM printer is characterized by higher overall costs.

This happens because Fortus 450mc has a larger working chamber, which allows it to work on larger batches, especially for smaller components (such as component 4) and to recover the high cost of materials. In fact, it permits to split energy and labor costs into several pieces, with a reduction in total costs, subdivided into individual components products. Additionally, the manpower contribution is less than that required by the other solutions; thus, it will occupy a shorter time interval, therefore leading to a generally lower labor cost.

Comparing the values of the Table 3 and Figure 8, it is possible to notice how in the case of the lone materials costs, the values were generally much less than the total. This is less evident for the Fortus 450mc, whereas it occurs above all for the EOS P396, whose labor and energy costs consequently have a large impact compared to the total: in fact, the process of filling the working chamber with inert gas (nitrogen) lengthens the cycle time even more, determining a lower production capacity and therefore a smaller number of pieces into which the various cost items are divided.

Immediately after the EOS P396, the printer that guarantees lower costs for building a component is the sPro 60: in fact, in 3 cases (for components 2, 5, and 6), the costs are very close to those of the EOS, whereas in the case of the component 4, it is the cheapest among the analyzed 3D powder bed fusion printers.

Following in increasing order of cost, there are then both the HP Jet Fusion 3D printers (versions 3200 and 4200), which follow the EOS P396 only for components 1 and 3, whereas they have higher costs than even the sPro 60 in other cases. Of course, last comes the Fortus 450mc, which, excluding case 4, always has the highest overall cost per component. As already mentioned, the high cost of the raw materials (in the form of filament) and the need to use an additional material to allow for the workpiece to be supported determines this unfavorable positioning in the costs classification.

Table 4 reports the values of the unit contribution margin (UCM), which represents the difference between the unit selling price and the unit variable costs, or the profit obtainable in a year by dedicating this printer exclusively to the production of one of the 6 components. Through a hypothesis, it is considered that it is possible to sell all the good built pieces at the set price. The selling price of the different components is assumed to be equal to four times the lowest cost to build it with one of the 3D printers analyzed.

For the same reasons expressed in the calculation of total costs, the highest UCM is always obtained for the SLS EOS P396 printer, except for component 4. This result is not surprising because the EOS was the 3D printer characterized by the lowest total costs among all those analyzed. Fortus 450mc is characterized by lower values, except for component 4. The behavior of the sPro and HP printers can best be described as intermediate.

In reality, having the highest UCM alone is no guarantee of greater profits if it is not also accompanied by a good production capacity and low waste rate and costs. Thus, a high margin does not guarantee good profits if the number of pieces over which it is distributed is low. Paradoxically, a minor UCM could lead to a greater profit if it is multiplied by a larger number of pieces produced.

**Figure 9:**
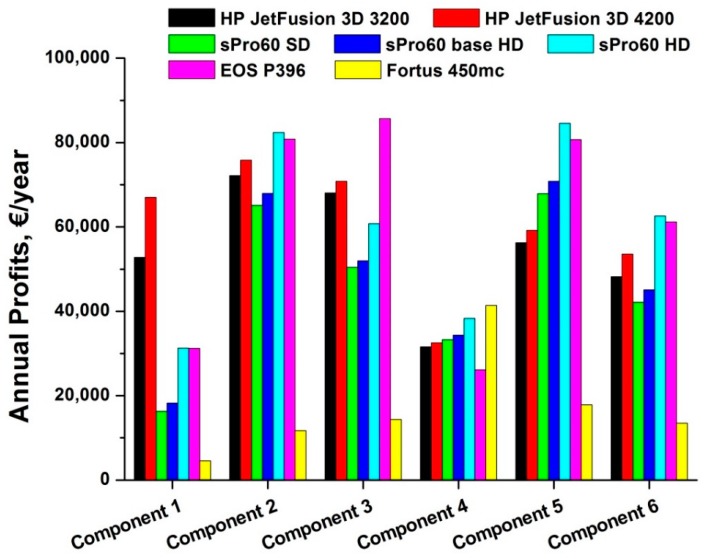
Bar chart of annual profits for each case study analyzed.

Figure 9 shows the profit values for each studied combination of 3D printer and produced component. A defect rate equal to 2% for all 3D printers was considered.

Observing Figure 9, it can be noticed that in the case of the production of component 1, working with an FDM technology printer generates a very modest annual profit compared with the other results (only € 4.576). This result is mainly due to two factors: a low margin (compared to what can be achieved with other 3D printers) due to a high cost for component, close to the selling price and to a very low production capacity related to component 1 (336 good pieces), which leads to comparable values of total costs and revenues.

The printers that are the most profitable for components 2, 5 and 6 are the selective laser sintering (SLS) technology ones, i.e., the EOS P396 and the sPro 60 HD (with values very close to each other). However, this result is due to very different factors: for the EOS P396, characterized by a good construction speed, but certainly not the highest, the determining factor was the total cost for component, due to the economic material PA 2200, whose low cost allows for one to make up for higher costs of both energy and manpower. In contrast, the other printer that uses the SLS technology, the sPro 60, allows for high profits for the three cited components thanks to a high construction speed and therefore a greater annual production capacity. This is especially true for the sPro 60 HD, which is the most advanced version.

The material used for sPro 60 (Duraform PA) is one of the most expensive materials on the market, in the category of polyamide 12 powders. However, the lack of auxiliary costs (for support material, chemical agents, etc.) makes the incidence of Duraform not excessively heavy in the calculation of total costs.

Instead, for components 1 and 3, the maximum profit is guaranteed by the HP Jet Fusion 3D 4200, thanks to its very high processing speed. After the sPro 60 HD, the HP 3D machines are the ones with the greatest speed and the greatest production capacity. In this manner, they make up for a high cost per component, mainly due to the cost of the chemical agents, “fusing agent” and “detailing agent”, and for a reduced working chamber (380 × 284 mm in plane), which is the smallest among the 3D printers studied. Thus, it is the one amongst those studied that best resembles a production machine.

Fortus 450mc is positioned at the end, since it is a machine that exploits a very different technology from the previous ones and is not yet suitable for a production area. The actual processing time is very high, especially due to the impossibility of working multiple objects at the same time, and consequently, the number of products produced is much lower than other printers. It allows for good profit results exclusively for component 4. The high cost for the FDM nylon 12 base material, to which the cost for the support material is added, is very important.

The economic analysis was performed by calculating the recovery period of the initial investment. The prices of selected 3D printers vary in the range of 100,000–400,000 €. This variability is essentially due to three factors: the technology, the processing speed and the accuracy in building the individual details of the object.

The EOS P396 is the one with the highest price, both because of the technology used (SLS) and for the high speed of construction. Furthermore, the minimum layer thickness achievable is 60 microns (it varies from 0.06 mm to 0.18 mm depending on the material used), making it one of the most accurate printers on the market.

The other SLS printer, the sPro 60, is priced slightly below the EOS, confirming the cost of this additive technology. In reality, the price of this printer is lower for the minor versions (sPro 60 SD and HD Base), but the performance also naturally decreases (and not on a small scale). The layer thickness varies in the range of 0.08–0.15 mm depending on the material used.

The prices of the HP Jet Fusion 3D 3200 and 4200 printers are approximately half that of the EOS P396 one, with a production capacity very close to those of the sPro 60. In addition, the use of chemical agents, which are added to the powder material, guarantees a high definition of the final product, with a layer thickness range of 0.07–0.10 mm, depending on the type of powder used.

The cheapest and most affordable printer, however, is the Fortus 450mc. The low purchase price is due to FDM technology, which is one of the most widespread types for additive manufacturing. Unfortunately, its filament extrusion process limits the achievable precision, with a layer thickness included in the range of 0.127–0.33 mm; thus, the minimum value is very high. Moreover, the Fortus 450mc construction speed (and in general, the speed of FDM technology) is certainly lower than that achievable using other AM processes (Carneiro, Silva, and Gomes 2015 [32]; Ligon et al. 2017 [33]).

Therefore, machines that lead to higher profits are those that have higher purchase prices, and conversely, those that yield low profits are the cheapest ones on the market. To assess which printer will be able to pay back its purchase cost thanks to its annual profits, two hypotheses have been considered: the first one is that the cost incurred for the purchase of the printer is fully supported by the company’s own capital, i.e., without bank loans; the second one is that discounting of future cash flows can be ignored, such that the profit generated in future years is worth the same as profit generated today.

**Figure 10:**
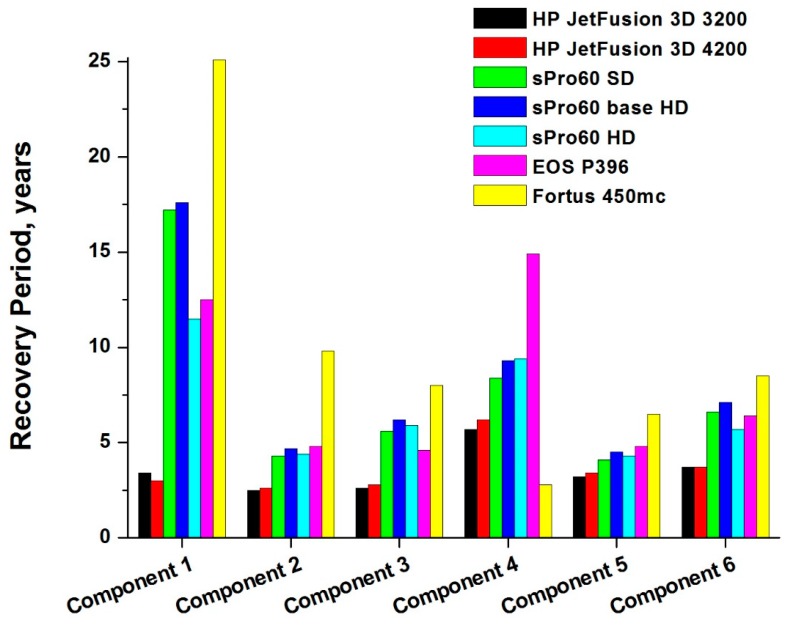
Recovery period of the initial investment.

Figure 10 presents the values of the recovery period for the initial investment in all the scenarios analyzed.

The two 3D printers that allow the recovery of their purchase cost in fewer years are HP Jet Fusion 3D 3200 and 4200. Despite the allowed profits not being the best, they are still sufficient to repay the competitive cost of purchase in a short time (on average, 3.6 years for the HP Jet Fusion 3D 4200 and 3.5 years for the 3200).

The initial investment weighs heavily on the EOS P396 printer, which turns out to be a much more expensive machine than the HP Jet Fusion 3D printer. Despite its high profits, the average recovery period is much longer than that of HP printers (approximately 8 years).

Good profits are also achievable by means of the different versions of the sPro 60 but not sufficiently high to repay in a short time the high cost of its purchase: the average of initial investment recovery years is approximately 6.9 years for the sPro 60 HD, 7.7 years for the sPro 60 SD and 8.2 years for the sPro 60 Base HD.

The worst results are obtained for the Fortus 450mc: the payback time is longer than 25 years in the case of component 1. In reality, however, the amount spent for its purchase is very small compared to the prices of other 3D printers considered, and it is therefore competitive if compared in general to the purchase price of a generic AM machine in the same performance range. The real problem is represented by the combination of a very low processing speed, which does not allow for it to obtain a good production volume, and very high production costs, which causes a low UCM value. These two factors together cause low profits, which do not allow for recovering the purchase cost over a few years.

### 3.2. Impact Evaluation

As part of the environmental impact analysis of the various additive manufacturing technologies, different categories of environmental impact must be considered (Le Bourhis et al. 2014 [34]; Huang et al. 2013 [35]):

- Global warming (or climate change);
- Reduction of ozone in the stratosphere;
- Photochemical ozone formation in the troposphere;
- Eutrophication;
- Acidification;
- Toxicity to humans;
- Eco-toxicity;
- Land use;
- Depletion of fossil fuels and mineral resources.

**Figure 11:**
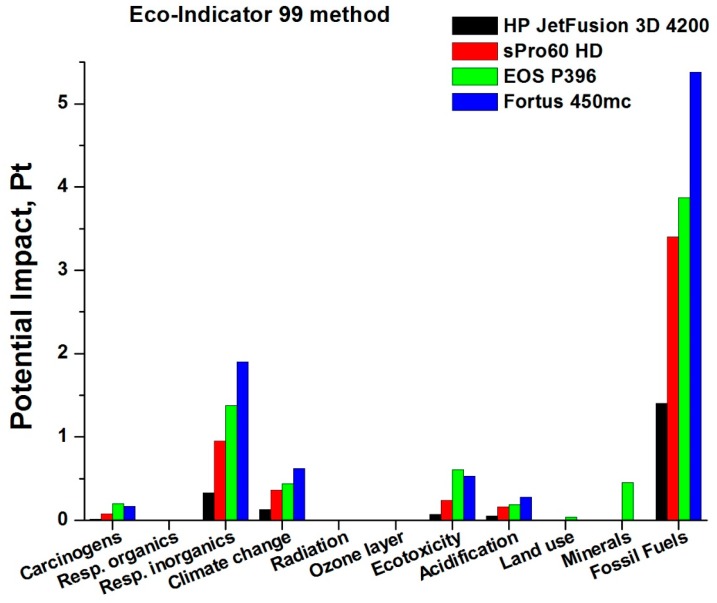
Normalized impact potentials for each 3D printer.

In Figure 11, the different impact potentials, divided into the different categories under examination, are reported. These values are reported for each of the four AM technologies analyzed. 

Analyzing Figure 11, is possible to note that the greatest potential impact is related to the consumption and depletion of resources, particularly fossil fuels, for each of the AM technologies analyzed. The damage caused to human health, including that from inorganic substances (to the respiratory tract) and that caused by climate change, is always relevant. The contributions related to toxic emissions (eco-toxicity) and those that modify acidity and nutrient levels (acidification and eutrophication), which worsen the quality of the ecosystem, should also not be overlooked. More importantly, it should be noted that there are considerable differences between the different AM technologies analyzed: the greatest impact potentials are associated with the Fortus 450mc (FDM) machine, then with the two selective laser sintering (SLS) machines, EOS P396 and sPro 60; finally, the technology characterized by the lowest impact potential is the multi-jet fusion (MJF), above all due to its much lower electricity consumption and therefore very limited resource consumption compared to other technologies.

**Figure 12:**
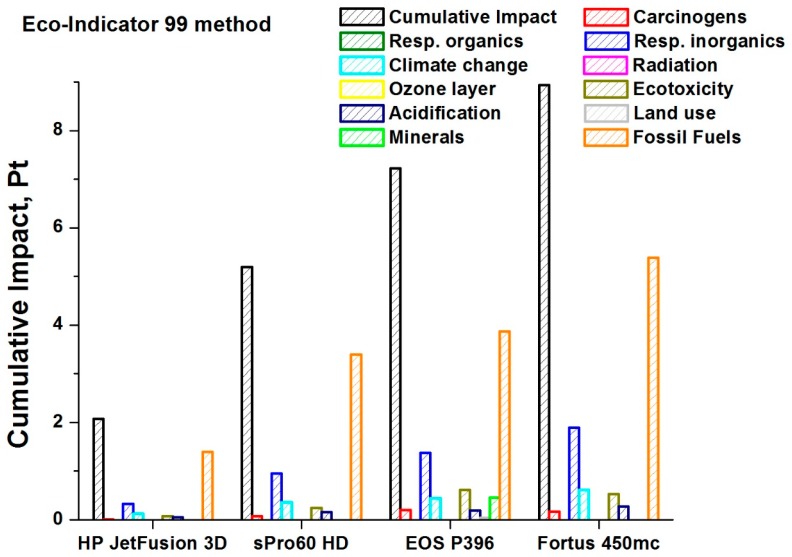
Cumulative potential impacts for each 3D printer.

Figure 12 summarizes what has been described up to now, using an overall impact potential, calculated for the various AM machines and technologies under examination.

Figure 12 trends confirm that the greatest cumulative impact is associated with the Fortus 450mc (FDM technology), which therefore turns out to be the technology with the lowest eco-compatibility among those analyzed. Instead, the lower potential is associated with the HP machine, based on the MJF technology, which is confirmed as the preferable from the point of view of environmental impact, due also to its smaller contribution associated with damage due to the effect of inorganic substances on the respiratory tract.

This result is therefore directly related to the electricity consumption, which, as described in the previous sections, is very high for the FDM machine compared to the other technologies in question.

In light of these assumptions, the results obtained are homogeneous in terms of the impact categories affected by the 4 machines. The most significant impact is due to the electricity consumption, especially for the Fortus 450mc, because of the larger number of hours required to perform the same process. On the other hand, the impact of the raw material used is much lower.

In general, the machine that has the greatest environmental impact is the Fortus 450mc, followed by the SLS machines, firstly EOS P396 and then sPro 60. The one that has the least negative effect on the environment is the HP Jet Fusion 3D 4200, whose cumulative impact is much less than half the values achieved by the SLS machines and much less than a quarter of the FDM technology values.

## 4. Conclusions

This work analyzed various additive manufacturing technologies in detail and compared them in terms of the realization of thermo-plastic material components. An overall analysis of both a technical-economic and an environmental nature was performed to obtain a clearer and more complete point of view about the considered technologies. The technologies that were analyzed in detail are selective laser sintering (SLS), fused deposition modeling (FDM), and the innovative HP multi-jet fusion technology (MJF).

It has been verified that regardless of the particular technology, at the present time, additive manufacturing still suffers from too long times for additive construction, especially for large components, which limits its application in practice to rapid prototyping.

The advantage of SLS or MJF technologies is associated with allowing for the realization of several components at the same time, thanks to the possibility of working on the entire construction surface by means of a laser or lamp-type thermal source, without a significant increase in sintering or melting time, compared to the case of a single component. For FDM technology, on the other hand, it is not possible to work on several pieces at the same time, leading to a considerable increase in processing times in the case of batches of several components. We must also consider that AM machines are characterized by small working chambers, and the dependence of the building speed of the object on the material used is still strong.

The economic analysis has highlighted how the Fortus machine, which employs FDM, is the one characterized by the lowest production capacity, with the highest total cost for the production of a single component inside the lot, because of the impossibility of a synchronous production of several components. In addition, the FDM machine is characterized by a lower UCM and lower profits for almost all the geometries that were analyzed in the present paper.

The analysis on the environmental impact, performed on a work cycle of the same batch of elementary components on the different machines, revealed that the technology that has the greatest impact on the environment is FDM, whereas the one characterized by minor effects is multi-jet fusion. The behavior of SLS machines is intermediate, above all due to the considerable consumption of fossil fuels, which are necessary for providing electrical energy to the production machine.

## Figures and Tables

**Table 1:** 3D printers and AM technologies analyzed.

Case Study	3D Printer	Print Technology
I	Fortus 450mc – Stratasys	FDM
II	EOS P396	SLS
III	sPro 60 HD – 3D Systems	SLS
IV	HP Jet Fusion 3D	MJF

**Table 2:** Powder quantity and weight of component 1 built by EOS P396.

Components (n°)	Powder Volume in the Chamber (mm^3^)	Powder Weight in the Chamber (kg)	Component Volume (mm^3^)	Component Weight (kg)
1	15,398,000	6.78	100,700	0.044
4	15,398,000	6.78	402,800	0.177

**Table 3:** Cost of materials for each component/printer case study.

Raw Material Cost (€/component)	HP JetFusion 3D 3200	HP JetFusion 3D 4200	sPro60 SD	sPro60 baseHD	sPro60 HD	EOS P396	Fortus 450mc
Component 1	8.33	8.33	9.96	9.96	9.96	4.24	26.50
Component 2	0.58	0.58	0.68	0.68	0.68	0.29	1.83
Component 3	0.75	0.75	0.84	0.84	0.84	0.39	2.33
Component 4	0.04	0.04	0.03	0.03	0.03	0.02	0.10
Component 5	1.31	1.31	1.52	1.52	1.52	0.67	3.07
Component 6	1.74	1.74	2.03	2.03	2.03	0.87	4.10

**Table 4:** Value of the unit contribution margin for each component/printer case study.

UCM (€)	HP JetFusion 3D 3200	HP JetFusion 3D 4200	sPro60 SD	sPro60 baseHD	sPro60 HD	EOS P396	Fortus 450mc
Component 1	32.72	32.95	28.52	28.85	30.19	35.27	14.29
Component 2	3.36	3.36	3.44	3.45	3.46	3.75	2.57
Component 3	4.99	5.00	4.84	4.85	4.88	5.62	4.19
Component 4	0.56	0.56	0.62	0.62	0.62	0.53	0.78
Component 5	8.51	8.53	9.38	9.39	9.44	9.99	8.82
Component 6	9.17	9.20	9.83	9.86	9.99	10.87	8.76
